# Pseudo-Placentational Endometrial Hyperplasia in the Bitch: Case Series

**DOI:** 10.3390/ani11030718

**Published:** 2021-03-06

**Authors:** Gabriele Marino, Alessandra Sfacteria, Giuseppe Catone, Antonina Zanghì, Fabiana Pecchia, Angela Difrancesco, Marco Russo

**Affiliations:** 1 Department of Veterinary Sciences, University of Messina, 98168 Messina, Italy; asfacteria@unime.it (A.S.); gcatone@unime.it (G.C.); zanghia@unime.it (A.Z.); pecchia.fabiana@gmail.com (F.P.); a.difrancesco88@gmail.com (A.D.); 2 Polivet, 00138 Rome, Italy; 3 Ambulatorio Veterinario Piccoli Animali da Compagnia Mariotti & Longhin, 39044 Egna, Italy; 4 Department of Veterinary Medicine and Animal Production, University of Naples, Federico II, 80137 Naples, Italy; marco.russo@unina.it

**Keywords:** dog, uterus, placenta, pyometra, histology

## Abstract

**Simple Summary:**

Pseudo-placentational endometrial hyperplasia is an uncommon lesion of the canine uterus. The lesion is characterized by a bizarre tissue organization resembling the layers of the mature maternal placenta. It may be inducible by foreign body insertion in the dioestrus uterus and probably encloses the mechanism of canine placentation. The ordinated proliferation may subvert to disorganized forms when the stimulus is biological and triggers an immune response. In this view, the pseudo-placentational endometrial hyperplasia may explain some unknown features of the cystic endometrial hyperplasia/pyometra complex. The report of six new spontaneous cases will help the knowledge and the clinicopathological framing of this unique lesion. Considering the physiological changes of endometrium in late dioestrus and early anoestrus, in the authors’ opinion, the pseudo-placentational endometrial hyperplasia term should be limited to the well-organized forms detectable by gross examination or ultrasound imaging.

**Abstract:**

Canine pseudo-placentational endometrial hyperplasia differs from the classical form of cystic endometrial hyperplasia for the well-organized tissue architecture resembling the canine placenta. After the discovery, it has been inconstantly reported. The present work reports the clinicopathological details of six spontaneous cases retrieved retrospectively from a large database. The lesion was found in young non-pregnant female dogs (median 2.0 years) at the end of dioestrus. It could be imaged by ultrasound and was always grossly detectable as single or multiple uterine enlargements of 2–3 cm in diameter with a villous whitish tissue growing on the mucosa and occluding the lumen. Histology confirmed the tissue architecture of the canine placenta with a basal glandular layer, a connective band, a spongy layer and a tortuous and compact labyrinth, often poorly recognizable. The pseudo-placentational hyperplasia is a non-inflammatory proliferative lesion although numerous mast cells inhabit the connective band, and a superimposed inflammatory infiltrate was seen in a case. Canine pseudo-placentational endometrial hyperplasia has very peculiar features, and it is a model for canine placentation and may help to better understand the cystic endometrial hyperplasia/pyometra complex.

## 1. Introduction

In canine species, cystic endometrial hyperplasia (CEH) is a common lesion, especially in elderly patients, and is characterized by hypofertility and potential evolution in pyometra. A second form of endometrial hyperplasia was discovered in 1914 during experiments of the surgical introduction of porcelain balls in the uterine lumen to induce contraception [1]. The experiment failed but some bitches developed endometrial reactions at the site of the surgical incision. The endometrium proliferated in an organized structure, resembling a juvenile placenta.

Similar but spontaneous lesions were observed in bitches presenting symptoms of pseudopregnancy at the time of hysterectomy [2,3]. The main contributors to the genesis of such lesion are those deriving from the experiments of Nomura and collaborators that induced various degrees of an endometrial decidual-like reaction, named deciduoma, inserting stainless steel wire, olive oil, silk suture, bouillon solutions with or without barium, uterine auto or allografts and a suspension of *Escherichia coli* [4,5,6,7,8,9]. Deciduoma was inducible only in early and mid-diestrus, under progesterone stimulation [10,11,12]. If the stimulus was biological, immune infiltration developed, and the cystic proliferation of deciduoma subverted as in CEH [8,9]. 

Two spontaneous cases of canine deciduoma have been occasionally found during surgery in young and asymptomatic female dogs in diestrus. The lesion was recognized by segmental distension of the uterus and histologically mimicked the layers of the canine placenta [13,14].

Considering that the term deciduoma indicates a tumor of decidual tissue, its use was discouraged soon after the revision of all the cystic conditions of the uterus of the female dog. The lesion was recognized as a form of endometrial hyperplasia that strictly resembles the endometrial changes occurring during canine placentation, and the definitive term of pseudo-placentational endometrial hyperplasia (PEH) was proposed [15,16].

Later, other three case reports of PEH [17,18,19] have been described. One case was an incidental finding during a routine ovariohysterectomy of a 9.5-month female dog with a history of cystitis and urolithiasis and at the end of diestrus [17]. A second case was diagnosed histologically after a non-conservative c-section, in a seven-year pregnant dog, although a non-well-recognizable placental remnant had been already identified by ultrasound from the 35th day of pregnancy [18]. Finally, in the third case (five years old), the lesion was identified and deeply described by ultrasound at the end of diestrus in a asymptomatic female dog [19]. The lack of previous descriptions of PEH did not allow differential diagnosis with hyperplasia and neoplasia of the uterus, and the dog underwent ovariohysterectomy allowing a definitive pathological diagnosis.

PEH seems a pure proliferative lesion, but an evident infiltration of immune cells may be detected [2,14,20]. As for CEH, an association of PEH and pyometra complex has been recently proposed and considered as an underestimated entity [21].

Based on this data, the causes and the occurrence of the spontaneous PEH still being unclear or debated, a retrospective study on a large clinicopathological database was designed finalized to the description of the clinical, gross and histopathological details of six new cases of spontaneous PEH.

## 2. Materials and Methods

Data were retrieved retrospectively from the clinicopathological database of the Veterinary Teaching Hospital (VTH) of Messina (Italy), in the period 1977–2020. They included female dogs which had undergone ovariohysterectomy, with organs available for pathological examination. This included fixation in 10% neutral buffered formalin, embedding in paraffin wax and staining with haematoxylin and eosin as a routine technique with more other stains if necessary.

Clinical data of each case were reviewed, including ultrasound reports and images, pre-surgical blood testing and progesterone levels, vaginal cytology and cardiological and anaesthesiology reports when available.

Data are presented as the median and range. To obtain epidemiological data, the PEH group was compared (age, breed and phase of the oestrus cycle) with all the cases of the database with the Mann–Whitney test for unpaired data and odds ratio setting the significance at *p* > 0.05.

## 3. Results

### 3.1. Epidemiology

A total of 662 female dogs (and uteruses) were revised retrospectively, and only six cases of PEH were found, with a prevalence of 0.9%. The evaluation of the phase of the oestrus cycle was carried out through reproductive history, clinical examination, vaginal cytology and progesterone assay. However, even in the partial absence of such data, the phase was confirmed by histological examination of the ovary and uterus. Five hundred and twenty-three dogs (79%) were in dioestrus as in all the PEH cases. However, the relation with the phase of the oestrous cycle was not supported by a significant odds ratio (*p* = 0.68). The median age of the population was 8.0 (range: 1–18) years while in the PEH group was 2.0 (range: 1–6). The *p*-value, calculated with the Mann–Whitney test for unpaired data of *t*-test, equaled 0.001, and this difference was statistically significant. The six cases (D1, D2, D3, D4, D5, D6) included two mixed, one Pinscher, one Labrador, one Sicilian hound and one Cairn terrier (Table 1), but no breed predisposition was found.

### 3.2. Clinical Findings

**Figure 1:**
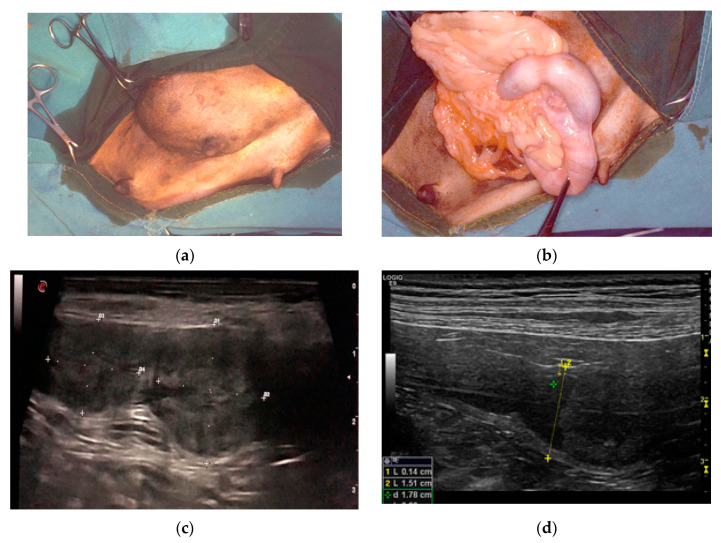
Clinical findings in three different cases of pseudo-placentational endometrial hyperplasia: (**a**) inguinal hernia with a uterine content in case D2; (**b**) at the opening of the hernia, the uterus with segmental swelling is evident; (**c**) ultrasound appearance of the lesion, resembling a placental remnant in case D4; D = distance (**d**) ultrasound appearance of the lesion in case D5, L= length, D = distance.

Clinical history of the PEH cases showed one or more episodes of pseudopregnancy (D1, D2) after previous heat. Case D1 showed again pseudopregnancy signs after removal of the genital tract. They were seen in heat 1.5 or 2 months before the admission and were not mated. The female dogs were asymptomatic with blood parameters into the normal ranges. Surgery was done for anticonception purposes (D1, D3, D6). Case D2 presented an inguinal hernia that at surgical correction revealed a uterine content (Figure 1a,b).

Ultrasound showed a focal widening of the uterine horn with two, ovoid, broadly base, pedunculated masses (case D4) protruding from the endometrium and filling most of the lumen. The lesions were heterogeneous in echotexture, slightly hyperechoic, and reminiscent of cerebral convolutions, with well-preserved wall layering (Figure 1c). In case D5, the uterus had a small amount of poorly particulate endoluminal fluid, which was more evident in the cranial part of the right horn, and a parenchymal endoluminal proliferation of about 1.5 × 2 cm in size. It showed an undulating hypoechoic crown with a thickening of 3 mm and an anechoic center (Figure 1d). Based on these findings, PEH was suspected in both cases. Lacking reproductive interest, ovariohysterectomy was performed.

### 3.3. Gross Pathology

**Figure 2:**
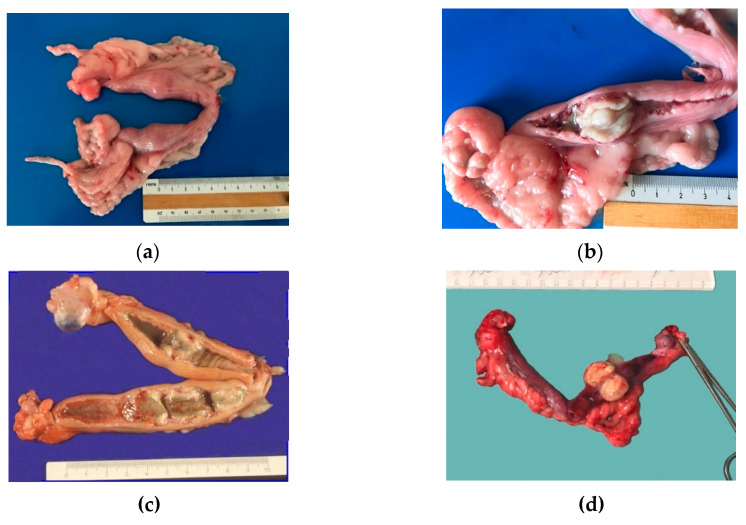
Gross findings in cases of pseudo-placentational endometrial hyperplasia: (**a**) the unopened uterus of case D1 with two spherical swellings; (**b**) the uterus of case D1 opened at the level of the swelling; the uterine mucosa is reddish; the lesion is almost white and seems leaned on the mucosa; (**c**) the genital tract of case D2 with four hyperplastic lesions which appeared greenish and accompanied by an abundant dirty fluid; (**d**) case D6 lesion with always the same size and appearance.

Grossly, the unopened uteruses had one (D3, D5, D6), two (D1, D4) or four (D2) ovoid distended areas, 2–3 cm in diameter, that resembled an early pregnancy (Figure 2a). Many longitudinal folds were present on the uterine surface. Corpora lutea were constantly seen in ovaries. Corpora lutea and cysts were detected in two cases (D2, D5).

On the cut section, the uterine mucosa was reddish and thickened, especially at the swelling level, where a soft white-greyish tissue was detected on the mucosa along with cloudy fluid in the lumen. The mass, impeding spontaneous drainage, caused modest fluid accumulation in case D2. The uterine mucosa in PEH appeared as covered by many yellowish-white villous protrusions that formed cylindrical bands occluding the lumen (Figure 2b–d).

### 3.4. Histopathology

**Figure 3:**
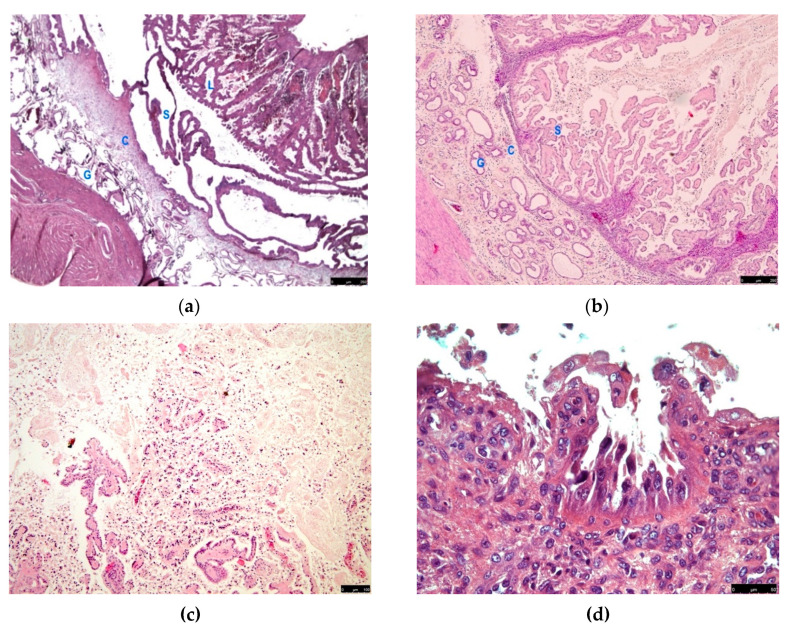

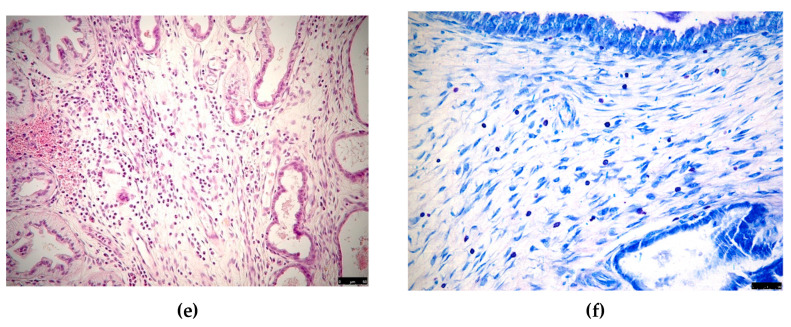
Histological findings in cases of pseudo-placentational endometrial hyperplasia: (**a**) the complete architecture of the lesion (D1) with 4 layers (G = glandular, C = connective, S = spongy, L = labyrinth), haematoxylin-eosin, bar = 250 μm; (**b**) the lesion (D2) with labyrinth zone degenerated, haematoxylin-eosin, bar = 250 μm; (**c**) case D5 dissolution of the inner layer for coagulative necrosis, haematoxylin-eosin, bar = 100 μm; (**d**) case D6, exfoliation of foamy cells in the inner layer, haematoxylin-eosin, bar = 50 μm; (**e**) inflammatory infiltrates in case D2, haematoxylin-eosin, bar = 50 μm; (**f**) numerous mast cells showing metachromasia in the connective layer of case D1, Giemsa stain, bar = 50 μm.

The described gross lesion showed histologically an endometrial proliferation forming an ordered structure recalling the layers of a complete canine placenta. A basal layer consisting of ectatic glands (glandular layer) was recognizable; a connective band sustained large elongated ectatic glands, separated by thin trabeculae of columnar epithelium, comparable to the spongy layer of decidua; an inner layer consisting of papilliferous tortuous and compact proliferation, lined by columnar epithelial elements with abundant cytoplasm, comparable to the compact layer of the decidua or labyrinth (Figure 3a,b). The smooth muscle layer in this segmental portion was thinner than other areas of the uterus.

In the luminal layer, numerous degenerative changes were found, such as cell exfoliation and coagulative necrosis. As a result, necrotic debris admixed with mucoid endometrial secretions partially replaced the inner layer in some cases (D2, D5, D6) (Figure 3c,d).

In all the cases, the coloration of Giemsa allowed the finding of a fair number of non-degranulated mastocytes, recognizable by the characteristic metachromasia, in all the layers but very abundant in the connective tissue band (Figure 3f). In case D2, a superimposed inflammatory infiltration was detected with neutrophils, eosinophils, lymphocytes and plasma cells (Figure 3e).

The uterus, in portions not affected by the lesion, had a thickened myometrium and a thick endometrial mucosa with a glandular epithelium in the active secretory phase (end of dioestrus). The superficial epithelium of the endometrium occasionally presented foamy cells organized in small papilliferous proliferations, similar in smaller scale to the spongy layer (PEH-like changes). Haemorrhage was found in the submucosa with red cells infiltrating the interstitial spaces.

The ovaries presented corpora lutea with vacuolized cells (end of dioestrus). Follicles were also present at various stages of growth. Paraovarian cysts were found in two cases (D2, D5).

## 4. Discussion

This study reported the clinicopathological features of six cases of PEH on 662 female dogs, estimating a prevalence of about 1%. A significant presence of PEH in young animals was found in agreement with other reports [13,14], although not exclusive of such age [17,18,19].

The phase of the oestrus cycle of PEH reports often overlaps with the pseudopregnancy period of female dogs. This may explain it because PEH has been referred to as the pseudopregnant uterus [2,3], speculating it was a component of pseudopregnancy [15]. Even in our series, two cases (D1, D2) had a history of clinical pseudopregnancy in previous heats, and one developed signs of pseudopregnancy again after the removal of the ovary. The experiments of Nomura have established that PEH is a reaction of the endometrium to physical stimuli in the mid dioestrus of pregnant and non-pregnant female dogs [10,11]. Moreover, a spontaneous case has been detected during pregnancy [18]. PEH has been always diagnosed around one to two months after oestrus [13,14,17,18,19]. It is likely that it grows in the dioestrus and starts to regress during the early anoestrus (the time of pseudopregnancy).

The female dogs affected by PEH are asymptomatic with blood parameters in the normal ranges [13,14,17,18,19]. However, endometritis and pyometra may superimpose in uteri affected by PEH and modify the clinical presentation [21]. Inguinal hernia with uterus protrusion (hysterocele) is quite common in dogs [22] and is generally associated with pyometra or pregnancy. PEH increasing the size of the uterus may facilitate the dislocation of the uterus, as observed in case D2.

Ultrasound examination may detect PEH [18,19], as reported in cases D4 and D5. If the spongy layer is predominant, hyperechoic linear striations perpendicular to the hyperechoic mucosal surface are seen [19]. PEH resembles a placental remnant or an involuting placental site and differential diagnosis may be difficult in course of pregnancy [18].

Considering that PEH is generally diagnosed at the end of dioestrus, the genital tract presents, grossly, typical features of this period such as serosal longitudinal folds, corpora lutea at the ovary and congested uterine mucosa [2,17]. In the unopened uterus, PEH is characterized by one or more 2–3 cm ovoid distended areas that may resemble placental sites [2,3,13,14,15,17,19]. On section, PEH is soft white-greyish tissue on the mucosa of the uterine swelling associated with cloudy fluid, debris or mucus [3,13,15,17,19]. PEH has been also reported in 0.5–1.5 cm exophytic nodular structures with a broad base of attachment or pedunculated [18]. Then, PEH is a grossly detectable lesion that arises from predetermined sites.

Although PEH may be strongly suspected by ultrasound and gross pathology, histology alone may reveal the typical tissue organization. The authors suggest using the term PEH only for well-organized spontaneous lesions, with three or four layers of the canine placenta (glandular, connective, spongy and labyrinth zone). The four-layer PEH, as observed in cases D1, D3 and D4, has been reported in limited reports [13,15,18]. The inner layer (labyrinth) often disappears for coagulative necrosis, and the spongy layer seems to dissolve into the lumen. The shadow of the layer reveals a previous presence, and the detachment has to be considered as an evolution of the lesion. This three-layer (glandular, connective, spongy and degenerated labyrinth zone) form of PEH has been more commonly reported [1,3,14,16,17,19] and was diagnosed in cases D2, D5 and D6.

Other papers reporting mild forms of PEH do not show the complete or incomplete tissue architecture but a lesion with a mild or moderate degree of spongy-like proliferations of the superficial epithelium of endometrium on a plus or minus separated and cystic glandular zone [2,4,5,6,7,8,9,10,11,12,20,21,23,24]. The superficial cells were columnar, often vacuolated and degenerated and very similar to decidual cells. These PEH-like changes or mild forms of PEH were also observed in the reported cases in the areas adjacent to the swellings and should be differentiated or considered among the features of the endometrium during the end of dioestrus and the early anoestrus [25,26,27].

The plane of separation of the canine placenta at the delivery lies in the spongy layer and the labyrinth is expulsed with the foetus and its membranes. The connective layer and the residual spongy layer degenerates in collagenous necrotic masses that progressively sloughed into the lumen while the endometrial glands of the glandular zone became normal in size and shape [25]. PEH likely follows the same destiny, and after its detachment, the endometrium turns back to the normal appearance. The materials sloughing down into the uterine lumen are compressed in cylindrical masses and expelled from the vulva [3]. There are no reports of PEH in mid or late anoestrus, where no PEH-like changes are visible on the uterine mucosa [2,25].

The pathological significance of PEH is probably low. Any kind of endometrial hyperplasia might be a cause of embryo resorption, then CEH or PEH may be found in female dogs with unexplained infertility or pregnancy loss [24]. PEH may cause an obstacle to drainage of fluid and facilitate endometritis as observed in case D2.

PEH, due to its close resemblance with the placenta, is likely to be a tissue showing an active role in modulating the local immunosurveillance. Despite the absence of evident immune cells, a large number of mast cells were detected in all the layers but very abundant in the connective tissue band. This population was observed in all the cases and has not been previously investigated in PEH or canine placenta. Only recently a resident population of mast cells has been investigated in canine uteri during the oestrus cycle [28]. However, placental mast cells have been studied in humans [29] where uterine-derived histamine produced by resident mast cells is considered a key regulator of implantation and decidualization. As surveillance cells, they can trigger, degranulating, a local inflammatory response. 

Chronic and acute endometritis may be concomitant to PEH [2,20], as observed in case D2. It is not known if PEH originates the inflammation or is simply invaded by a diffuse phlogistic reaction. As for CEH, a parallel association of PEH and pyometra can be seen in female dogs [21]. There is evidence that CEH is a disorganized form of PEH while pyometra and pregnancy have many parallelisms, starting from the same mechanism of compartmentation [1,8,9]. Probably we are not describing different pathologies or different physiological mechanisms but the same phenomenon with a different fate.

## 5. Conclusions

PEH is an uncommon gross lesion exclusive of the canine uterus, and it may be diagnosed in postpubertal animals from the dioestrus to the early anoestrus. The tissue architecture incredibly recalls all the layers of the mature canine placenta, although mild forms may be frequently observed. PEH is the expression of the reactivity of the uterus to foreign bodies, embryos or germs.

## Figures and Tables

**Table 1:** Clinicopathological findings of the cases of pseudo-placentation endometrial hyperplasia (PEH). P4 = progesterone.

*n*	Clinical	Gross	Histology
D1	Mixed Breed, 1.5 years; history of recurrent clinical pseudopregnancies; in heat 2 months before without mating; asymptomatic; routine anticonception surgery	Longitudinal folds on the uterine serosa; 2 ovoidal uterine swellings in the left and right uterine horn; whitish lardaceous lesions at the swelling: hyperemic uterine mucosa, corpora lutea	4-layer PEH at swelling level; PEH-like changes on the remaining uterine mucosa; corpora lutea with degenerated lipid-filled luteal cells confirming end of diestrus
D2	Mixed Breed, 6 years; no pregnancies but history of recurrent clinical pseudopregnancies; in heat 2 months before without mating; inguinal hernia; neutrophilia; end of diestrus (P4 = 1 ng/mL); ovariohysterectomy and herniorrhaphy	Longitudinal folds on the uterine serosa; 3 ovoidal uterine swellings in the left uterine horn and 1 in the right; greyish lardaceous lesions at the swelling; hyperemic uterine mucosa, dirty fluid in the lumen; corpora lutea and a large cyst in the right ovary	3-layer PEH at swelling level; PEH-like changes on the remaining uterine mucosa; diffuse endometritis; corpora lutea with degenerated lipid-filled luteal cells; paraovarian cyst
D3	Pinscher, 2 years; in heat 1.5 months before without mating; asymptomatic; vaginal cytology compatible with diestrus; routine ovariohysterectomy	One ovoidal uterine swelling in the left uterine horn; whitish lardaceous lesion at the swelling; slightly hyperemic uterine mucosa, corpora lutea	4-layer PEH at swelling level; PEH-like changes on the remaining uterine mucosa; corpora lutea with active luteal cells confirming diestrus
D4	Sicilian hound, 2 years; in heat 2 months before with mating; no pregnancy; end of diestrus (P4 = 1 ng/mL); at ultrasound uterus widening (2 cm) for two adjacent endoluminal mass lesions with suspicion of PEH or placental remnants; ovariohysterectomy	Longitudinal folds on the uterine serosa; 2 ovoidal uterine swellings in the left uterine horn; greyish lardaceous lesions at the swelling; hyperemic uterine mucosa; corpora lutea	4-layer PEH at swelling level; PEH-like changes on the remaining uterine mucosa; corpora lutea with degenerated lipid-filled luteal cells
D5	Labrador, 5 years; 2 previous pregnancies; in heat 2 months before without mating; end of diestrus (P4 = 1 ng/mL); fluid-filled uterus; at ultrasound uterus widening (2 cm) for an endoluminal mass lesion with suspicion of PEH; ovariohysterectomy	Longitudinal folds on the uterine serosa; one ovoidal uterine swelling in the right uterine horn; whitish lardaceous lesion at the swelling; hyperemic uterine mucosa, mucous fluid in the lumen; corpora lutea and a large cyst in the right ovary	3-layer PEH at swelling level; PEH-like changes on the remaining uterine mucosa; corpora lutea with degenerated lipid-filled luteal cells; paraovarian cyst
D6	Cairn terrier, 10 months; in heat 1.5 months before without mating; asymptomatic; diestrus (P4 = 20 ng/mL); routine ovariohysterectomy	Longitudinal folds on the uterine serosa; one ovoidal uterine swelling in the right uterine horn; whitish lardaceous lesion at the swelling: hyperemic uterine mucosa, corpora lutea	3-layer PEH at swelling level; PEH-like changes on the remaining uterine mucosa; active corpora lutea

## Data Availability

The data presented in this study are available on request from the corresponding author.
